# Epidemiological Profile of HPV Genotypes Among Moroccan Women with High-Grade Squamous Lesions and Invasive Cervical Cancer from Souss-Massa Region

**DOI:** 10.3390/tropicalmed10030061

**Published:** 2025-02-26

**Authors:** Hanane Rogua, Laila Ferrera, Nezha El Mansouri, Mohamed Nejmeddine, Said Chouham

**Affiliations:** Laboratory of Cell Biology and Molecular Genetics, Department of Biology, Faculty of Sciences Agadir, Ibn Zohr University, Cite Dakhla, Agadir P.O. Box 8106, Morocco

**Keywords:** HPV, genotypes, epidemiology, cervical cancer, HSIL, Morocco

## Abstract

The aim of this study was to investigate the distribution of human papillomavirus (HPV) genotypes among cervical cancer cases in Moroccan women living in the Souss-Massa region. A total of 155 formalin-fixed, paraffin-embedded cervical tissue samples were tested for the presence of HPV DNA using a semi-nested PCR assay. HPV genotypes were identified using a direct Sanger sequencing assay. The prevalence of HPV was 85.8%. HPV DNA was found in 87.5% of high squamous intraepithelial lesions (HSIL) cases and 85.7% of invasive cervical cancer (ICC) cases. Ten distinct HPV genotypes were identified, including seven high-risk HPV (HR-HPV) genotypes and three low-risk HPV (LR-HPV) genotypes. Among HR-HPV genotypes, HPV16 was the most prevalent in both HSIL and ICC, detected, respectively, in 42.9% and 55.6% of cases. In ICC cases, HPV18 was the second most common genotype detected, in 10.3% of cases. In addition, HPV31, 33, 35, 45 and 58 were detected in 10.4% of ICC cases. LR-HPV genotypes, namely HPV62, 70 and 87, were detected in 2.4% of ICC cases. Adenocarcinoma (ADC) accounted for 4.1% of ICC cases, with HPV 16 and HPV 18 identified in 60% and 40% of these cases, respectively. Overall, our findings show that the genotypes covered by the bivalent and nonavalent HPV vaccines account, respectively, for 65.4% and 74.6%. These results highlight the importance of introducing HPV vaccination and primary HPV testing for mass screening in Morocco in order to effectively prevent and manage cervical cancer and ultimately save women’s lives.

## 1. Introduction

Despite advances in cervical cancer screening and treatment, the disease remains a global public health problem. In 2020, there were approximately 604,000 new cases and 342,000 deaths from cervical cancer worldwide, making it the fourth most frequently diagnosed cancer and the leading cause of cancer-related death in women [1,2].

According to GLOBOCAN predictions for the year 2040, the incidence of cervical cancer should decrease in Europe, North America, Oceania and Asia, with reductions ranging from −29% to −1.3%. By contrast, Latin America and the Caribbean will register an increase in incidence of 4.2%. The most concerning situation is predicted for Africa, where a substantial increase of 89% is expected [2].

In Morocco, cervical cancer remains a major public health problem, ranking as the second most common cancer in women, in terms of both incidence and mortality. According to GLOBOCAN estimates for 2020, there were around 2165 new cases and 1199 deaths from cervical cancer in Morocco [2]. In order to diagnose precancerous lesions and cervical cancer at an early stage, the National Cancer Prevention and Control Plan (NCPCP) between 2010 and 2019 prioritized cervical cancer screening in women aged 30–50 every three years using visual inspection with acetic acid and Lugol iodine in five regions, enabling the institutionalization of an organized cervical cancer early detection program [3,4]. With its second edition for the period 2020–2029, in line with the World Health Organization’s global campaign to eliminate cervical cancer as a public health problem by 2030, the NCPCP program prioritized the inclusion of HPV vaccination in the national vaccination program for girls aged 11, the introduction of HPV genotypic surveillance, in addition to extend the current screening program to all regions of the country, and to restructure and reorganize the program with a view to improve screening participation to 70% [4].

Persistent human papillomavirus (HPV) infection, particularly with high risk-HPV (HR-HPV) genotypes, is recognized as the main cause, although not sufficient in itself, of the development of cervical cancer [5,6]. The global HPV prevalence is estimated around 10–12% worldwide. However, the prevalence of HPV vary considerably between less developed countries and more developed ones [7,8]. In addition, the distribution of its genotypes varies considerably among women in different countries and regions, even within the same country [9]. In general, HPV16 is the most common HR-HPV genotype worldwide, while the prevalence of other genotypes tends to differ between geographical regions [10].

It is imperative to conduct studies on the prevalence of HPV, the distribution of its genotypes and their relationship with cervical cancer in each country and in its different regions. These studies are essential to assess the relevance of genotyping in cervical cancer screening and treatment, enlighten vaccination policies and clarify future medical decisions and vaccination strategies.

To date, no studies have been conducted on women with cervical cancer in Southern Morocco. Therefore, the aim of this study was to investigate the distribution of HPV genotypes in cervical cancer cases of Moroccan women living in Souss-Massa region.

## 2. Materials and Methods

### 2.1. Sample Collection

This was a descriptive, quantitative, retrospective study including 157 formalin-fixed, paraffin-embedded (FFPE) cervical tissues of women with a histological diagnosis of high-grade squamous lesions (HSIL) and invasive cervical cancer (ICC) between January 2019 and July 2022 in private and public anatomy and pathological cytology facilities in Agadir. Five to eight sections of FFPE samples were collected in “nuclease-free” Eppendorf tubes.

### 2.2. DNA Extraction

Samples were deparaffinized by triple treatment with xylenes and then washed with ethanol at decreasing concentrations at room temperature. DNA extraction was carried out using the commercial PureLink Genomic DNA Mini Kit (Thermo Fisher Scientific, Waltham, MA, USA) following the manufacturer’s instruction. Then, an amplification of a 110-base-pair (bp) fragment of the β-globin housekeeping gene was performed using PC03/PC04 primers to assess DNA integrity and the absence of PCR inhibitors in all extracted DNA [11].

### 2.3. HPV Detection End Genotyping

HPV DNA presence was assessed by semi-nested PCR using three consensus primers: MY09/MY11 and GP6+ [12]. PCRs were performed in a final volume of 20 µL, consisting of 1X DreamTaq Green PCR Master Mix (Thermo Fisher Scientific), 4 pmol MY09 primer, and 12 pmol MY11 and GP6+ primers. For each reaction, both a negative control (using Milli-Q water) and a positive control (a sample containing previously detected and confirmed HPV DNA) were incorporated [6].

The PCR program consisted of four steps: an initial denaturation step of 5 min at 94 °C; followed by 15 amplification cycles, each with 30 s denaturation at 94 °C, 30 s primers annealing at 55 °C and 30 s extension at 72 °C; then 35 amplification cycles, each with 30 s denaturation at 94 °C, 30 s primers annealing at 50 °C and 30 s extension at 72 °C; and finally, an elongation step of 7 min at 72 °C.

If positive for HPV DNA, PCR products were sequenced using the Applied Biosystems Sanger sequencing kit following the manufacturer’s instructions as previously detailed [13].

## 3. Results

### 3.1. Study Population Characteristics

After amplification of a fragment of the β-globin gene, two samples tested negative and were therefore excluded from the study. Women whose samples were included in the study ranged in age from 20 to 81 years, with an average age of 54.6 ± 12 years. Of the 155 samples included in this study, the ages of 17 patients were not available from their medical records.

### 3.2. Histological Analysis Results

The histopathological results of the 155 included samples reveals that 5.2% of patients had HSIL (n = 8) while 94.8% were affected by ICC (n = 147). The histological analysis of ICC cases indicated that 61.2% were moderately-differentiated invasive squamous cell carcinomas (SCC) (n = 90), 17% were well-differentiated invasive SCC (n = 25), 7.5% were poorly differentiated invasive SCC (n = 11), 10.2% were invasive SCC (n = 15), while 4.1% were adenocarcinoma (ADC) (n = 6).

### 3.3. Overall HPV DNA Prevalence

PCR results indicated the presence of viral DNA in 85.8% of samples (133/155). The prevalence of viral DNA in samples according to pathological status was 87.5% for HSIL (7/8) and 85.7% for ICC (126/147). In ICC cases, HPV DNA was detected in 83.3% of ADC (5/6) and 85.8% of SCC (121/141).

### 3.4. Overall HPV Genotypes Distribution

Direct sequencing analysis revealed that 78.2% of cases (104/133) contained only one type of HPV (sHPV). In 6% of positive cases (8/133), the sequences overlapped, indicating the coexistence of multiple HPV types (mHPV). Meanwhile, in 21 samples, sequencing did not produce any readable sequence; thus, they were labeled as an uncharacterized HPV genotype (HPVx). Of samples containing sHPV, 97.1% were HR-HPV (101/104), while only 2.9% were low risk-HPV (LR-HPV) (3/104).

**Figure 1:**
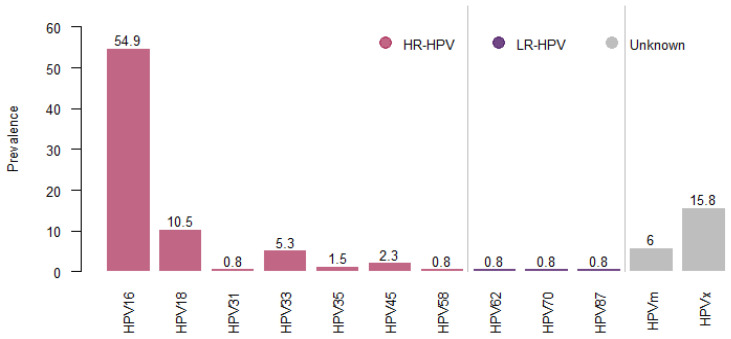
HPV genotypes distribution among Moroccan women with HSIL and ICC from Souss-Massa region. HR-HPV: high-risk HPV, LR-HPV: low-risk HPV, mHPV: multiple HPV, HPVx: uncharacterized HPV.

The distribution of genotypes in the HPV-positive samples is shown in Figure 1. Overall, ten distinct HPV genotypes were detected: 7 HR-HPV genotypes and 3 LR-HPV genotypes. Of the HR-HPV genotypes, HPV16 was the most prevalent, detected in 54.9% of cases (n = 73), followed by HPV18, detected in 10.5% of cases (n = 14). In addition, HPV33 was detected in 5.3% of cases (n = 7), HPV45 in 2.3% of cases (n = 3), HPV35 in 1.5% of cases (n = 2), and HPV31 and 58 each in 0.8% of cases (n = 1). On the other hand, LR-HPV genotypes, HPV62, 70 and 87, were detected in 0.8% each (n = 1).

### 3.5. Distribution of HPV Genotypes According to Histopathological Results

The distribution of genotypes according to histopathological profile reveals that 42.9% of HPV-positive HSIL samples (n = 7) were associated with HPV16 (n = 3). The most prevalent genotypes in HPV-positive ICC samples (n = 126) were HPV16, detected in 54.33% of cases (n = 69); HPV18 was detected in 9.47% (n = 12), HPV33 in 5.51% (n = 7) and HPV45 in 2.36% cases (n = 3) (Table 1). In HPV-positive ADC samples (n = 5), two genotypes were detected: HPV16 (60%) and HPV18 (40%).

## 4. Discussion

Understanding the distribution of HPV genotypes in cervical cancer can facilitate the implementation of an effective vaccination program, anticipate the potential outcomes of HPV genotype-specific vaccines and screening tests, and improve our understanding of the oncogenic potential of different HPV genotypes. To date, no research has been conducted in the southern region of Morocco on this specific population. Therefore, the aim of this study was to determine the prevalence and distribution of HPV genotypes in women with cervical cancer from the Souss-Massa region.

The histopathological analysis revealed that ICC was the predominant form, accounting for 94.8% of cases, with adenocarcinoma being detected in only 4.1% of them, and HSIL in 5.2% of cases, which was in line with previous investigations in Morocco [14,15]. In a study conducted in Casablanca by Meftah el khair and colleagues, it was noted that among SCC cases, moderately differentiated invasive SCC were the most common, followed by well-differentiated invasive SCC and poorly differentiated invasive SCC [14]; this result is consistent with our findings.

Our findings demonstrated the presence of HPV DNA in 85.8% of the samples. These results are consistent with those of earlier investigations conducted in other regions of Morocco, where the prevalence ranged from 62% [16] to over 90% [5,15]. Comparable results have also been reported in various other regions, such as Tunisia (83%) [17], Brazil (84%) [18], China (88.2%) [10], Congo (89%) [19], Gabon (91.4%) [20], and Algeria (92.7%) [21].

In our investigation, we found that of the HSIL samples, 87.5% were HPV positive and 42.9% of them were HPV16 positive. This is consistent with previous research reporting HPV16 as the predominant genotype in HSIL, detected in 47% [19,22].

Regarding ICC cases, HPV16 appeared to be the most prevalent HR-HPV genotype, detected in 55.6% of cases. This result is consistent with the findings of various studies conducted in Morocco, although the prevalence varies between studies from 45% [16] to 87.29% [15].

We identified HPV18 as the second most frequent genotype, in 10.3% of ICC cases. In Morocco, HPV18 has consistently been described as the second most common genotype [5,14,15], which is consistent with previous studies from around the world [18,23]. However, in studies conducted in Congo and Gabon, the HPV18 genotype was ranked third after the HPV33 genotype [19,20], while in China it was ranked third after the HPV58 genotype [10].

HPV infection is a necessary cause of cervical cancer, but in some cases, HPV DNA may not be detectable. This can occur due to factors such as the type of sample analyzed; indeed, FFPE samples often contain more fragmented DNA compared to fresh biopsies or frozen samples, making PCR amplification more challenging [24]. Additionally, when HPV integrates into the host genome, it may lose fragments of its DNA, such as the L1 gene, which is commonly targeted by PCR primers. which can prevent detection by the primers used in the study [25].

In a retrospective cross-sectional study including 10 575 ICC cases, HPV16 and 18 genotypes were identified in 71% of cases. A further 10% of cases were attributed to HPV31, 33, 35, 45, 52 and 58 [26]. Our data are consistent with these findings, as we detected the presence of HPV31, 33, 35, 45 and 58 genotypes in 10.4% of ICC. However, it should be noted that HPV52 was not detected in our study. This suggests that HPV52 may either have a weaker association with cervical cancer in our population or be one of the genotypes not identified by direct sequencing (mHPV and HPVx).

Our findings indicate that LR-HPV were detected in only 2.4% of cases, highlighting the rarity of the involvement of LR-HPV genotypes in ICC, as documented in the literature [26,27].

The HPV70 genotype, detected in one case of SCC in our study, has also been documented in two studies of HIV-positive women in Morocco [6,28]. Although HPV70 is classified as LR-HPV, its potential role in the development of cervical cancer in people with immunodeficiency diseases cannot be excluded.

In our current investigation, we identified HPV in 83.3% of ADC cases, which is consistent with global findings, spanning from 62.8% to 100% [20,29,30]. Among the HPV-positive ADC cases, we observed that HPV16 and HPV18 genotypes were present in 60% and 40% of cases, respectively, a pattern consistent with numerous studies. For instance, in Gabon, HPV16 and HPV18 were found in 75% and 25% of ADC cases, respectively [19]. In Congo, HPV16 was detected in 69.2% of cases, and HPV18 identified in 7.7% of ADC cases [20]. Similarly, in Greece, HPV16 was present in 73.08% of cases, while HPV18 was detected in 35.90% of ADC cases [29]. In addition, HPV45 has also been reported in ADC in many previous studies [11,26,30], but not in our study, probably due to the small number of cases analyzed.

To date, three safe and effective vaccines against HPV infections are used as a primary prevention strategy to reduce the risk of cervical cancer worldwide: the bivalent vaccine (Cervarix^®^) targeting HPV 16 and 18; the quadrivalent vaccine (Gardasil-4^®^) targeting HPV 6, 11, 16, and 18; and the nonavalent vaccine (Gardasil-9^®^) targeting HPV 6, 11, 16, 18, 31, 33, 45, 52, and 58 [31]. Our findings showed that the bivalent and quadrivalent vaccines each cover 65.4% of the genotypes identified among the studied population, while the nonavalent vaccine covers 74.6% of them.

Co-infection with several HPV genotypes is frequently reported in epidemiological studies. HPV genotypes may interact or show synergistic effects, potentially influencing the development or progression of cervical lesions. In addition, multiple infections have been associated with reduced 5-year survival in cervical cancer [18]. In our study, we observed mHPV infections in 6% of the cases. This prevalence is in line with similar studies in Brazil (4%) [18], and Algeria (10.2%) [21].

This study has some limitations. First, we were unable to obtain information on the FIGO stage of the samples due to restrictions on access to the Agadir regional oncology/radiotherapy center during and after the COVID-19 pandemic. Thus, we were unable to evaluate the distribution of HPV genotypes according to the severity of the pathology. Second, the use of direct sequencing for genotyping did not allow us to identify HPV types in samples with multiple infections. We need to improve this aspect further by using more robust and specific new generation sequencing methods, or tests designed to target a broad spectrum of genotypes.

## 5. Conclusions

This is the first study reporting HPV genotypes distribution among Moroccan women with cervical cancer from Souss-Massa region. The genotypes covered by the bivalent/quadrivalent and nonavalent HPV vaccines account, respectively, for 65.4% and 74.6% of cases. Our results show that introducing HPV vaccination in Morocco could offer a significant opportunity to reduce the burden of cervical cancer in the country. Given the high burden of this cancer in the country, the implementation of a primary HPV test for mass screening should be investigated. In addition, developing an indigenous HPV detection kit for cervical cancer screening could further reduce costs and improve accessibility in resource-limited settings. Such strategies are worth exploring in the Moroccan context.

## Figures and Tables

**Table 1:** HPV genotypes distribution by histopathological categories.

HPV Genotype	Histopathological Categories Type	
	HSIL (n = 7) % (n)	ICC (n = 126) % (n)
HPV16	42.9 (3)	55.6 (70)
HPV18	14.3 (1)	10.3 (13)
HPV31	-	0.8 (1)
HPV33	14.3 (1)	4.8 (6)
HPV35	-	1.6 (2)
HPV45	-	2.4 (3)
HPV58	-	0.8 (1)
HPV62	14.3 (1)	0.8 (1)
HPV70	-	0.8 (1)
HPV87	-	0.8 (1)
mHPV	14.3 (1)	5.6 (7)
HPVx	-	16.7 (21)

HSIL: high squamous intraepithelial lesions, ICC: squamous cell carcinoma, mHPV: multiple HPV, HPVx: uncharacterized HPV.

## Data Availability

The original contributions presented in this study are included in Supplementary Materials. Further inquiries can be directed to the corresponding author.

## Supplementary Materials

The following supporting information can be downloaded at: https://www.mdpi.com/article/10.3390/tropicalmed10030061/s1, File S1: Raw data.

## Abbreviations

The following abbreviations are used in this manuscript:

HPV	human papillomavirus
HR-HPV	high-risk HPV
LR-HPV	low-risk HPV
sHPV	single HPV
mHPV	multiple HPV
HPVx	uncharacterized HPV
FFPE	formalin-fixed, paraffin-embedded
HSIL	high squamous intraepithelial lesions
ICC	invasive cervical cancer
SCC	squamous cell carcinomas
ADC	adenocarcinoma
NCPCP	National Cancer Prevention and Control Plan
